# Ultrasound-enhanced fine-needle aspiration biopsy improves yield of solid benign parotid gland tumor tissue: a pilot study

**DOI:** 10.1186/s41747-026-00707-0

**Published:** 2026-04-02

**Authors:** Mira Naukkarinen, Yohann Le Bourlout, Minna Rehell, Jetta Kelppe, Kristofer Nyman, Jaana Rautava, Sanjeev Ranjan, Gösta Ehnholm, Jouni Rantanen, Kenneth P. H. Pritzker, Jussi Tarkkanen, Katri Aro, Timo Atula, Heikki J. Nieminen

**Affiliations:** 1 Department of Otorhinolaryngology–Head and Neck Surgery, University of Helsinki and Helsinki University Hospital, Helsinki, Finland; 2 Medical Ultrasonics Laboratory (MEDUSA), Department of Neuroscience and Biomedical Engineering, Aalto University, Espoo, Finland; 3 HUS Diagnostic Center, HUSLAB, Department of Pathology, Helsinki University Hospital and University of Helsinki, Helsinki, Finland; 4 Department of Radiology, HUS Diagnostic Center, University of Helsinki and Helsinki University Hospital, Helsinki, Finland; 5 Department of Oral and Maxillofacial Diseases, University of Helsinki, Helsinki, Finland; 6 Departments of Laboratory Medicine and Pathobiology, Surgery, University of Toronto, Toronto, ON Canada

**Keywords:** Biopsy (fine-needle), Biopsy (large-core needle), Parotid gland, Salivary gland neoplasms, Ultrasonography

## Abstract

**Objectives:**

Fine-needle aspiration biopsy (FNAB) and core-needle biopsy (CNB) are common methods to evaluate the pathology of an abnormal tissue mass. However, both methods have their limitations, *e.g*., FNAB samples often remain inadequate, and CNB is more invasive. A novel device, ultrasound-enhanced fine-needle aspiration biopsy (USeFNAB), can collect samples with greater cellular content quantitatively without compromising tissue quality. The purpose of this prospective study was to evaluate USeFNAB for the first time in humans *in vivo*.

**Materials and methods:**

This is a pilot study without any deviation from the contemporary management of the salivary gland tumor. Ten adult patients with a solid benign parotid gland tumor diagnosed by FNAB or CNB. Before parotidectomy, the tumors were sampled under ultrasound guidance with three different needle sampling techniques: USeFNAB, FNAB, and CNB. The influence of USeFNAB on the quantity and quality of the samples was investigated and compared with FNAB and CNB.

**Results:**

The quality of the cytological slides and histological tissue samples was similar in all samples obtained by USeFNAB, FNAB, and CNB. With USeFNAB, the mass increased on average by 1.6 and 3.4 times and the histological sample area by 1.7 and 3.4 times, as compared to FNAB and CNB, respectively.

**Conclusion:**

The results of this study demonstrated that USeFNAB seems to be a feasible and safe biopsy technique under *in vivo* conditions. In solid benign parotid gland tumors, USeFNAB increases sample yield as compared to conventional needle sampling methods, without affecting sample quality.

**Relevance statement:**

USeFNAB could improve the diagnostic accuracy of needle biopsies and facilitate ancillary techniques such as molecular diagnostic studies.

**Trial registration:**

Institutional permissions for the study were granted. The USeFNAB protocol went through risk analysis and mitigation for approval by the Finnish Medicine Agency (FIMEA) as an investigational device for research purposes (FIMEA/2023001788).

**Key Points:**

- USeFNAB showed an improved sample yield in solid benign parotid gland tumors compared to FNAB and CNB, without affecting sample quality.
- USeFNAB seems to be a feasible and safe biopsy technique.
- USeFNAB is a promising tool for improving the diagnostic accuracy of needle biopsies.

**Graphical Abstract:**

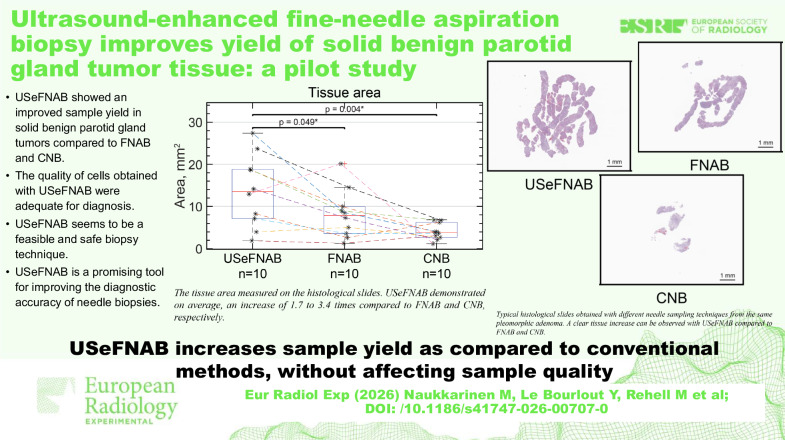

## Background

Needle biopsy sampling is a commonly used method to help determine the pathology of an abnormal tissue mass. An adequate needle biopsy sample allows the pathologist to identify all relevant diagnostic characteristics of the lesion, guiding the clinician to make optimal treatment decisions, ultimately leading to improved patient outcomes [1]. Common needle sampling procedures include fine-needle aspiration biopsy (FNAB) and core-needle biopsy (CNB).

However, both FNAB and CNB have their shortcomings. FNAB is notably characterized by a high rate of inadequate samples and false benign results [2–7]. CNB, by virtue of utilizing larger gauge needles, is more invasive, requires more effort to obtain, and is associated with a higher risk of complications [8–11], which may necessitate additional preprocedural planning and postprocedural monitoring. Additionally, CNB is not always a feasible biopsy modality, *e.g*., when the lesion is proximate to large blood vessels or nerves, posing significant challenges in ensuring patient safety and procedural success.

**Fig. 1:**
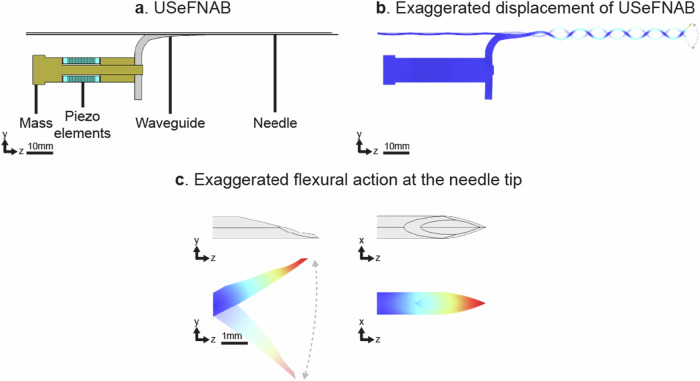
Visualization of the USeFNAB device. **a** Schematic representation of components of the USeFNAB’s handpiece. **b** Illustration of the device’s exaggerated displacements during operation. **c** Detailed view of the flexural displacement at the needle tip, intended to enhance the sample collection during the biopsy procedures. USeFNAB, Ultrasound-enhanced fine-needle aspiration biopsy

A recently introduced technique known as ultrasound-enhanced fine needle aspiration biopsy (USeFNAB) aims to obtain better FNAB samples by including tissue fragments akin to those obtained through CNB [12–14]. USeFNAB uses flexural ultrasonic actuation (f ≈ 30 kHz) at the hypodermic needle tip. This controlled bending displacement (< 100 μm), *i.e*., transversal-like movement with respect to the long axis of the needle, utilizes the mechano-acoustic effects of the needle to detach cells and/or tissue constructs from the target area [13, 14] (Fig. 1). Previous studies, using *ex vivo* animal and *ex vivo* human tissues, have shown that USeFNAB can collect 2‒6 times more tissue yield than CNB or FNAB, while maintaining comparable tissue quality [12–16]. In a clinical setting, such improvements could potentially offer more confident diagnostic conclusions through the sample being better representative of the sampled target and reduce the need for repeat biopsies, optimizing both radiological and clinical workflow, and patient experience.

One common target for imaging-guided FNAB is a salivary gland mass [17–19]. Achieving an accurate diagnosis of such a mass can prevent unnecessary surgical interventions for non-neoplastic lesions and asymptomatic benign neoplasms, and the extent of surgery for neoplasms can be planned adequately.

Based on these premises, the present study is the first study to evaluate the *in vivo* application of USeFNAB in human subjects, with a specific focus on solid benign parotid gland tumors. The primary aim of this prospective study is to evaluate the technical feasibility and performance of the method in a clinically relevant setting. The study compares USeFNAB to conventional FNAB and CNB in terms of tissue yield (mass and area), sample quality (cytological and histological), and diagnostic adequacy. For technical comparison of the performance, the dwells are limited to a single biopsy per method per tumor. Furthermore, procedural safety is evaluated to explore the potential of USeFNAB as a viable and minimally invasive alternative for diagnostic practice.

## Materials and methods

### Patients and tumors

For our prospective study group, 11 patients undergoing surgery for a parotid gland tumor were enrolled in the Department of Otorhinolaryngology–Head and Neck Surgery, Helsinki University Hospital, Helsinki, Finland, between September 2023 and January 2024.

The inclusion criteria were as follows: adult patients (over 18 years of age) who had a preoperatively diagnosed solid benign parotid gland tumor (primarily pleomorphic adenoma) either with FNAB or CNB and were scheduled for parotidectomy. The diameter of the parotid gland tumor had to be at least 15 mm on imaging to ensure that the tumor was sufficiently large for needle sampling with all three different methods.

The exclusion criteria were as follows: patients with a cardiac pacemaker or another electrical implant; patients with established heart disease, such as coronary artery disease, heart failure, or arrhythmia; and patients with severe immunodeficiency, minimizing the potential risks associated with the experimental device’s electricity and ultrasonic vibrations. The demographics and preoperative examinations of all 11 patients were retrospectively reviewed.

With one patient, the calibration failed during the procedure, causing the USeFNAB to not activate. This patient was excluded, and thus, our final study group consisted of 10 patients. Of them, 9 (90%) were female and 1 (10%) was a male. The mean age of the patients was 50.5 years (median 52, range 21‒78).

### Sampling

Each parotid gland tumor was sampled with all three needle sampling techniques: USeFNAB, FNAB, and CNB. The comparison was primarily between two different fine-needle techniques: USeFNAB and FNAB. CNB served as a reference method as it is also widely used in clinical practice. For each subject, the sequence of the biopsy methods was randomized prior to the procedure. The Excel (Microsoft, Redmond, WA, USA) function RAND was used to generate a uniformly distributed random number for each method. These numbers were then sorted in ascending order to define the sampling sequence. The samples were taken by an experienced head and neck radiologist (K.N.) with the help of an assistant (M.N. or M.R. and Y.L.B.) in the operating room with the subject under general anesthesia, immediately before the parotidectomy. The same radiologist took all the samples in a sterile manner. Ultrasound imaging guidance (HS60, Samsung, Soul, South Korea) was used for all sampling techniques during the whole biopsy procedure. In this study, a one-insertion per needle sampling technique was implemented to minimize the number of needle insertions and to have a technical comparison of the biopsy method. After taking the needle samples, surgery on the parotid gland tumor was performed according to the standard protocol.

#### USeFNAB

**Fig. 2:**
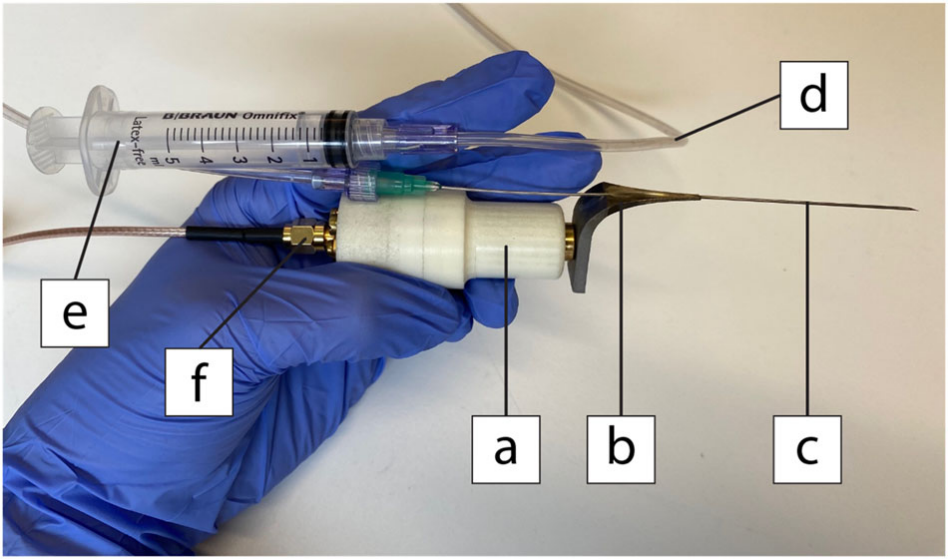
Photograph of the handpiece of the USeFNAB. The white enclosure contains the Langevin transducer (**a**) that produces a longitudinal oscillation that yields an operation frequency 30.86 ± 0.72 kHz (*n* = 10). Coupled to it is the waveguide (**b**) that converts and amplifies longitudinal to flexural oscillations. Connected to it is the 21-G hypodermic needle (**c**). Low pressure is controlled by an assistant using the extension hose (**d**) and syringe (**e**). The handpiece is connected to the main unit by the electrical cable (**f**). USeFNAB, Ultrasound-enhanced fine-needle aspiration biopsy

The USeFNAB system consists of a standard hypodermic needle (21-gauge, 120 mm; model 466564/3,100 STERICAN, B Braun) coupled with a waveguide which was connected to a Langevin transducer (nominal frequency 30 kHz) (Fig. 2). The waveguide was a three-dimensional printed piece of surgical grade EOS stainless steel 316 L (3D formtech OY) that converts and amplifies the longitudinal oscillation of the transducer to a flexural displacement at the needle tip [13]. The transducer was connected to the main unit, containing a wave signal generator (Analog Discovery 2, Digilent, Inc.) and an amplifier (Mariachi Oy). The main unit was controlled by a laptop and custom-made software.

Before the biopsy, the waveguide and needle construct were assembled in a sterile manner. A sterile hypodermic needle was connected to an autoclaved waveguide using a biocompatible adhesive (SmartBond® Adhesive, Orthodontics, Gestenco International AB). The distal side of the needle extended by 55 mm from the end of the waveguide. The biocompatible adhesive was allowed to solidify for at least 30 min prior to the biopsy.

**Fig. 3:**
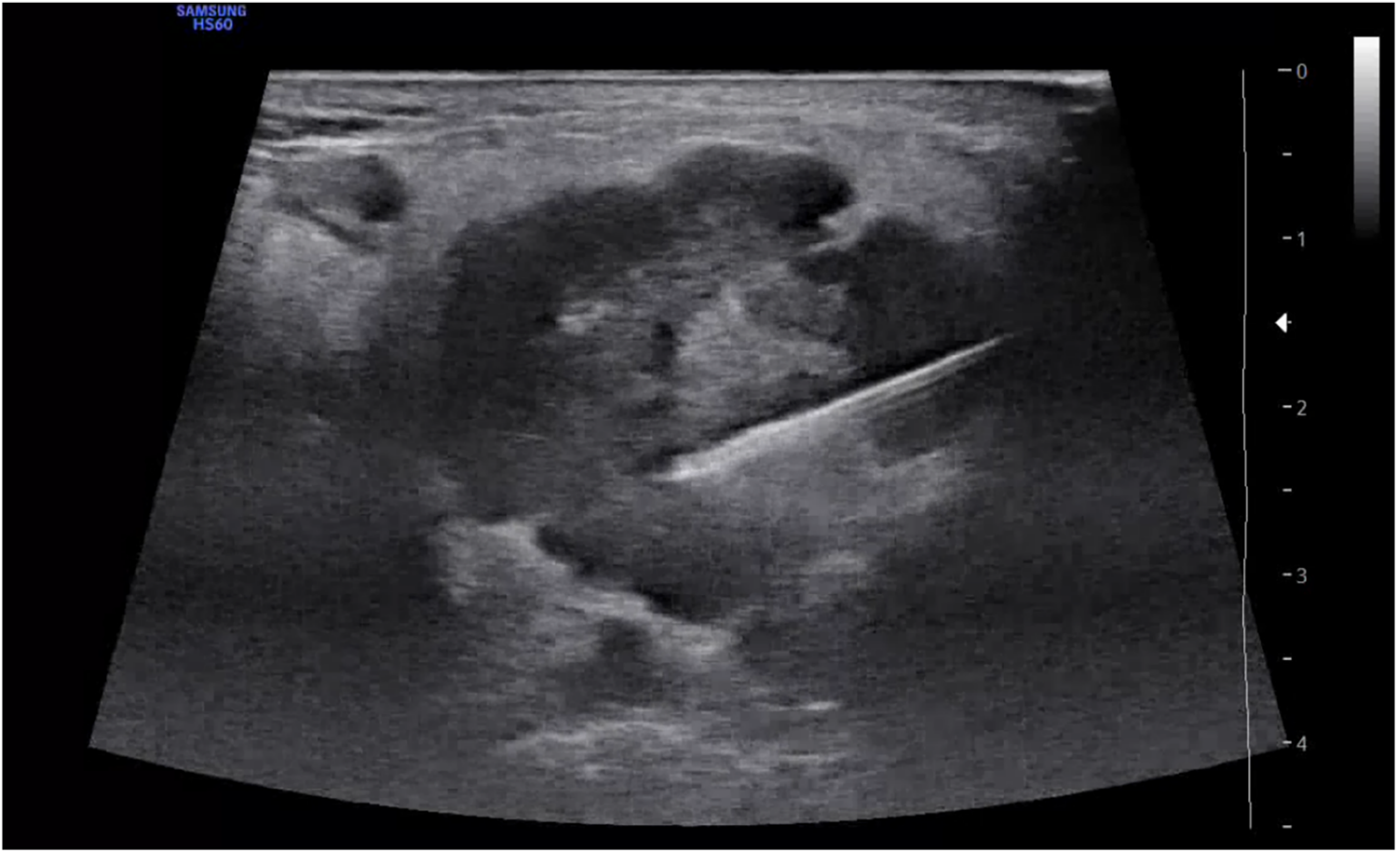
Ultrasound image obtained during the USeFNAB procedure with the Samsung HS60 device. The image distinctly shows the tip of the needle in the parotid gland tumor (pleomorphic adenoma) without artifacts or noise induced by USeFNAB. This confirms the device’s compatibility with current ultrasonic imaging modalities. USeFNAB, Ultrasound-enhanced fine-needle aspiration biopsy

Before the sampling procedure with USeFNAB, the needle and the waveguide were weighed using a precision scale (Kern ADJ 200-4, mass range: 400 mg–210 g, Kern). The waveguide was attached to the transducer, and the needle was connected by a pressure-rated catheter extension hose (BD, Franklin Lakes) to a 5-mL syringe (Omnifixâ Luer-Lock Solo, B Braun) to allow application of low pressure. The needle was inserted into the tumor under ultrasound guidance by the radiologist (Fig. 3). Before each sampling, the device was calibrated at low acoustic power (< 100 mW forward electric power) with the needle tip in the tumor. The resonance frequency was selected within this frequency range (30‒33 kHz), and the targeted time-averaged consumed electric power was set for 0.5 W. Records of the USeFNAB procedure showed a resonance frequency of 30.86 ± 0.72 kHz (mean ± SD; *n* = 10) and an average time consumed power of 0.39 ± 0.06 W (mean ± standard deviation; *n* = 10). After calibration, low pressure was applied up to the 3 mL mark of the syringe, and the wave generator began to produce the desired ultrasonic sequence for 10 s. Simultaneously, the radiologist moved the tip of the needle in a normal fan-like manner, back and forth (approximately 1‒2 cm) as well as laterally (approximately 7° between the stokes) at a rate of 1 movement per second, with the needle inside the tumor. After 10 s, the needle movement was stopped, the low pressure was released, the needle was removed from the parotid gland, and the needle and waveguide were weighted to assess the extracted mass. The collected sample was expressed into a container with 70% ethanol using the positive pressure of the syringe.

#### FNAB

FNAB was performed with a similar hypodermic needle (21-gauge, 120 mm) and 5-mL syringe as used for USeFNAB. The needle was connected by a hose to the syringe. The procedure was done in a sterile way and in a similar manner to USeFNAB. The weight of the needle was measured on the precision scale both before and after sampling to determine the sample mass. The sample was emptied into a container with 70% ethanol using the positive pressure of the syringe.

#### CNB

CNB was taken using the BioPince™ Ultra Full Core Biopsy Instrument (18-gauge, 100 mm; Argon Medical Devices). The throwing length of the CNB instrument was adjusted to 13 mm, which yields 9 mm specimen length. The tip of the BioPince needle was inserted into the tumor under ultrasound guidance, after which the sample was taken in a routine and sterile manner with only one hit from each tumor. The weight of the collected sample was measured using the precision scale. Finally, the sample was fixed in formalin.

### Adverse events

Immediate complications were assessed: First, the samplers and the surgeon visually examined the skin immediately after sampling. Second, the surgeon assessed during the operation whether any hematoma or other damage to the tumor or the surgical area could be observed.

Late complications were defined as those appearing after leaving the operation room up to 7 days after surgery. The patients’ records were reviewed, and each patient was called 1 week after surgery and asked how they had recovered from the needle sampling and surgery, if they had any symptoms and whether they had been in contact with any healthcare units due to any symptoms.

### Preparation of samples

All samples were processed at the Department of Pathology, Helsinki University Hospital, according to a standard clinical protocol.

#### FNAB and USeFNAB

The ethanol-fixed FNAB and USeFNAB samples were poured through a filter paper, and tissue fragments were picked for histological paraffin block preparation. The tissue fragments were dehydrated, cleared with xylene, and embedded in paraffin wax. Paraffin-embedded samples were cut into thin slices of 3‒4 µm using a microtome and then stained with hematoxylin-eosin for histological assessment. The remaining cell solution was centrifuged, placed on glass slides, dehydrated, fixed, and stained with a Papanicolaou stain for cytological assessment.

#### CNB

The samples fixed in formalin were dehydrated, cleared with xylene, and embedded in paraffin wax. Paraffin-embedded samples were cut into thin slices of 3‒4 µm using a microtome and then stained with hematoxylin-eosin for histological assessment.

### Analysis of samples

USeFNAB, FNAB, and CNB samples were evaluated by experienced head and neck pathologists (J.K. and Ja.Ra.). They analyzed the needle samples without knowing the biopsy modality associated with the sample or each other’s judgments. Afterward, in case of discrepancy between evaluations, a joint re-evaluation of the sample was performed to reach a consensus.

#### Cytology

From the centrifuged glasses, pathologists evaluated the quality, quantity, crushing artifact, and coagulation of each sample. The quantity of the cytological samples was scored as follows: 0 = nothing/empty slide or 1 = some tumor cells/adequate tumor cells. The quality of cytological samples was scored as follows: 0 = nondiagnostic (degeneration or any loss of morphologic features) or 1 = diagnostic (intact, representable cells). The crushing artifact and coagulation of each sample were evaluated with a yes or no rating.

#### Histology

Pathologists evaluated the histological quality of the samples in four different classes: 1 = 0‒25% of the tissue was intact, 2 = 26‒50% of the tissue was preserved intact, 3 = 51‒75% of the tissue was preserved intact, and 4 = 76‒100% of the tissue was preserved intact. By definition, an intact sample was considered when significant tissue degradation, coagulation, or unfavorable fragmentation affecting diagnostic assessment was absent. The diagnostic assessment of the biopsy sample was compared with that of the surgical resection specimen. If both concluded on the same diagnosis, the biopsy sample was considered adequate.

#### Tissue area

To remove bias from blood in the weighting measurement, an investigation of the average tissue area of each sample from the microscope slide was conducted. Scanned histological slides were analyzed with QuPath (open source software for digital pathology image analysis. version: 0.5.0) to determine the area of tissue on the slide using the threshold function (pixel color-based threshold) [20]. This area was then divided by the number of repetitions/cutovers on the slide (number of repetitions varying from 1 to 5).

### Statistical analysis

The statistical comparison of the groups with quantitative metrics was conducted in Matlab (release 2024 A, TheMathWorks, Inc.) using the Wilcoxon signed rank test (α = 0.05, two-tailed). Effect sizes of the paired differences were reported using the matched-pairs rank-biserial correlation.

## Results

Table 1 shows patient and tumor characteristics. All patients had undergone preoperative imaging (ultrasound or magnetic resonance imaging). The size of the parotid gland tumor was based on radiological imaging, and the measured diameter of tumors varied between 16 and 35 mm (mean 20.8 mm). A preoperative FNAB or CNB sample had been taken from all tumors before parotidectomy and had been diagnosed as solid benign parotid gland tumors (primarily pleomorphic adenomas).

**Table 1:** Patient and tumor characteristics

Patient number	Gender	Age (years)	Side	Size of the tumor by imaging (mm)	Pre-study sampling method	Pre-study diagnosis	Final histopathology
1	F	51	R	19	CNB	Myoepithelioma or PA	Myoepithelioma
2	F	58	R	22	FNAB	PA	PA
3	F	53	L	19	CNB	PA	PA
4	F	30	L	17	FNAB	PA	PA
5	F	49	R	18	FNAB	PA	PA
6	F	62	L	19	CNB	PA	PA
7	F	46	R	18	FNAB	PA	PA
8	M	78	L	16	CNB	PA	PA
9	F	21	L	25	CNB	PA	PA
10	F	57	L	35	FNAB	PA	PA

*CNB* Core-needle biopsy, *F* Female, *FNAB* Fine-needle aspiration biopsy, *L* Left, *M* Male, *PA* Pleomorphic adenoma, *R* Right

### Needle samples

FNAB and USeFNAB samples contained tissue construct and single cells, yielding cytological samples with histological fragments. The CNB sample did not include cytology but provided tissue construct, thus resulting in only a histological sample.

#### Histological quality

**Fig. 4:**
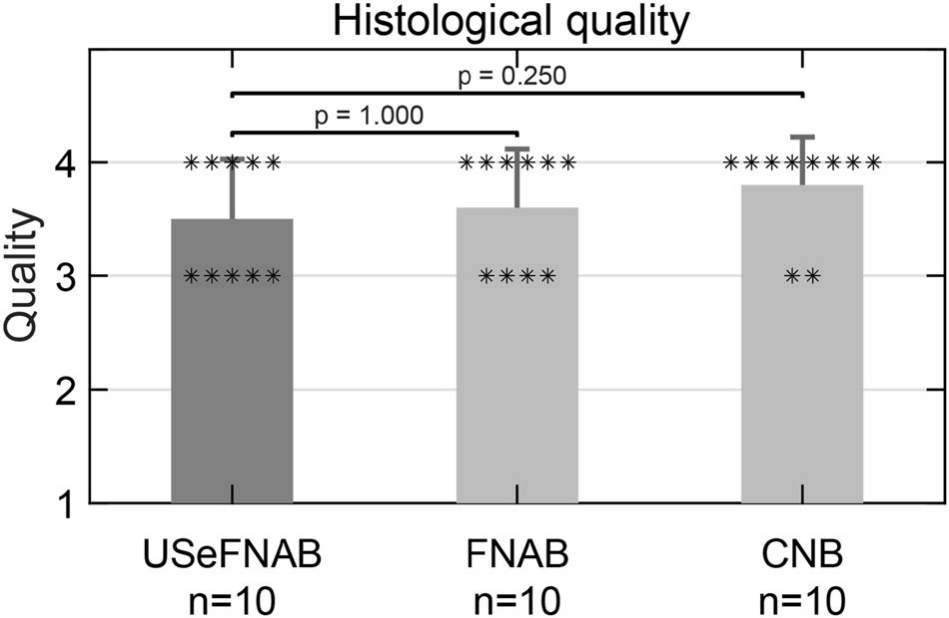
Histological quality assessed by the pathologists. The bars display the mean of histological sample quality of the biopsy samples (*n* = 10 per group), while the error bars indicate the standard deviation. Quality levels 1, 2, 3 and 4 refer to 0‒25%, 26‒50%, 51‒75% or 76‒100% of the sample being intact. USeFNAB provided similar histological quality as compared to the state-of-the-art methods. USeFNAB, Ultrasound-enhanced fine-needle aspiration biopsy

The assessment from the pathologists demonstrated average quality scores of 3.5 ± 0.5 (mean ± standard deviation) for USeFNAB, 3.6 ± 0.5 for FNAB, and 3.8 ± 0.4 for CNB (Table S1). No statistically significant differences were observed between the methods (0.25 ≤ *p* ≤ 1). The mean score difference between USeFNAB and FNAB was -0.10 ± 0.32 (mean ± standard deviation; Rank-biserial correlation *r*_*rb*_ = -1.00; *p* = 1.000) and between USeFNAB and CNB was -0.30 ± 0.48 (mean ± standard deviation; Rank-biserial correlation *r*_*rb*_ = -1.00; *p* = 0.250) (Table S1). The results indicated that the sample quality obtained with the different methods was comparable (Tables S1 and S2). Histological tissue samples prepared from USeFNAB, FNAB, and CNB material accurately represented the tumor when compared with the histology of the excised tumor, and all samples were considered adequate for diagnostic (Fig. 4, Table S2).

#### Cytological adequacy

Pathologist assessment on the cytological slides of FNAB and USeFNAB from each tumor showed adequate material for diagnostic evaluation. Additionally, there were no significant crushing artifacts or coagulation in any of the samples. All cytological preparations were evaluated as diagnostic by the pathologists.

#### Sample mass

**Fig. 5:**
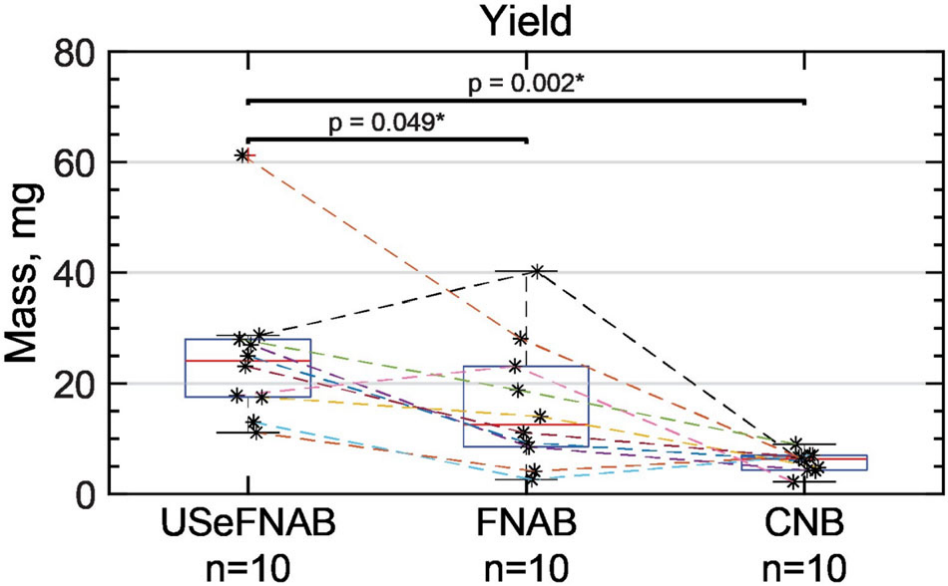
Measured mass of the biopsy specimens. The central mark shows the median, the box indicates the 25th to the 75th percentile, while the whiskers indicate the minimum and maximum non-outlier values; outliers are displayed with a red cross (*n* = 10 per group). The data points obtained from the same patient are connected with dashed lines. USeFNAB demonstrated a statistically significant higher tissue mass collected compared to standard FNAB (*p* = 0.049) or CNB (*p* = 0.002). CNB, Core-needle biopsy; FNAB, Fine-needle aspiration biopsy; USeFNAB, Ultrasound-enhanced fine-needle aspiration biopsy

The mean mass of the biopsy specimens was as follows (*n* = 10 per group, Fig. 5): USeFNAB: 25.24 ± 14.08 mg (mean ± standard deviation; *n* = 10); FNAB: 15.98 ± 11.74 mg; and CNB: 5.77 ± 1.90 mg. The results indicated that USeFNAB provided statistically higher yield compared to FNAB (mean difference ± standard deviation = 9.26 ± 12.41 mg; Rank-biserial correlation r_rb_ = 0.71; *p* = 0.049) or CNB (19.47 ± 13.97 mg; r_rb_ = 1.00; *p* = 0.002) (Table S3).

#### Tissue area

**Fig. 6:**
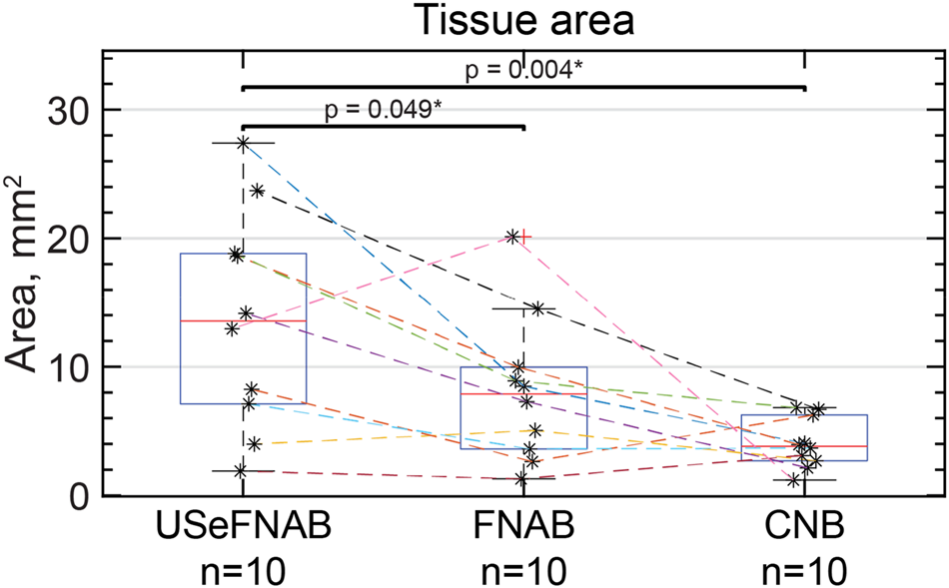
The tissue area on the histological slides measured with QuPath. The central mark shows the median, the box indicates the 25th to the 75th percentile, while the whiskers indicate the minimum and maximum non-outlier values; outliers are displayed with a red cross (*n* = 10 per group). The data points obtained from the same patient are connected with dashed lines. USeFNAB demonstrated a statistically significant increase compared to the state-of-the-art methods. On average, USeFNAB samples displayed 1.7 to 3.4 times more tissue compared to FNAB and CNB, respectively. CNB, Core-needle biopsy; FNAB, Fine-needle aspiration biopsy; USeFNAB, Ultrasound-enhanced fine-needle aspiration biopsy

**Fig. 7:**
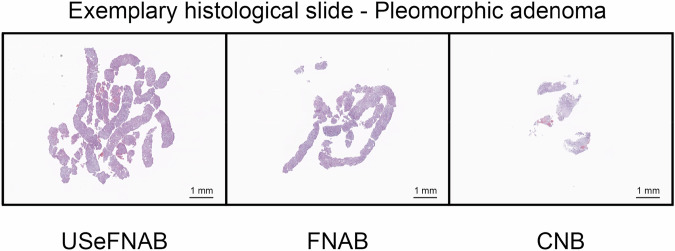
Typical histological slides obtained with different needle sampling techniques from the same pleomorphic adenoma of the parotid gland. A clear tissue increase can be observed with USeFNAB compared to FNAB and CNB. CNB, Core-needle biopsy; FNAB, Fine-needle aspiration biopsy; USeFNAB, Ultrasound-enhanced fine-needle aspiration biopsy

The average tissue area on the histological slides was as follows (*n* = 10 per group, Fig. 6): USeFNAB: 13.69 ± 8.47 mm^2^; FNAB: 8.19 ± 5.73 mm^2^; and CNB: 4.06 ± 1.95 mm^2^. The results indicated that USeFNAB provided statistically higher tissue area on the histological slide as compared to FNAB (mean difference ± standard deviation = 5.50 ± 7.13 mm^2^; Rank-biserial correlation r_rb_ = 0.71; *p* = 0.049) or CNB (9.63 ± 7.94 mm^2^; r_rb_ = 0.96; *p* = 0.004) (Table S4). Figure 7 shows typical histological slides obtained with different needle sampling techniques.

#### Needle tract

In two tumor resection specimens, a needle tract was visible. However, it was not possible to define which technique produced the tract. No tumor seeding along the needle tract was detected by the pathologists in either of the samples.

### Patient outcome

Immediately after needle sampling, no visible signs of tissue damage in the sampling area were observed in any of the patients. During parotidectomy, a minor hematoma was seen in one patient, but otherwise, no adverse events were observed during parotidectomy either.

One week after needle sampling and parotidectomy, all patients reported normal postparotidectomy symptoms, such as some pain and swelling in the surgical area. Seven out of ten patients were in contact with our department due to these symptoms. Antibiotic treatment was prescribed to two of the patients. Transient facial nerve dysfunction was observed postoperatively in three out of ten patients by the surgeon. However, no cases of permanent facial nerve paralysis were detected.

## Discussion

This is the first study in humans evaluating the USeFNAB technique *in vivo*. USeFNAB collected more material for pathological analysis than conventional FNAB and CNB, while maintaining intact tissue essential for accurate pathological assessment. The results suggest that USeFNAB is both feasible and safe in a clinical setting, thus providing a promising tool and potential to enhance diagnostic accuracy.

USeFNAB method induces an ultrasonic flexural oscillation at the tip of the needle [13]. The flexural oscillations with typical transverse displacements of tens of micrometers with respect to the needle center axis create shear forces at an ultrasonic frequency. This causes the needle bevel to detach tissue and cells from the mass of interest. Consequently, the method increases the quantity of tissue collected during the biopsy. In a recently published *ex vivo* human tissue study, USeFNAB collected 2.3 times more tissue yield than FNAB, without compromising sample quality [14].

For this study, solid salivary gland tumors were included. The tumors were preoperatively regarded as benign adenomas, predominantly pleomorphic adenomas. They were chosen as the first application of USeFNAB because FNAB is widely used in the diagnosis of salivary gland masses. Moreover, salivary glands provide a rather uniform series of common tumor types, allowing for effective comparison between conventional needle biopsy methods. Additionally, pleomorphic adenoma is the most common salivary gland tumor type [21, 22] and shows heterogeneous histological structure with a mix of tissue textures ranging from gelatinous mucin to firmer epithelial cells.

The bore volume of the 18-gauge CNB needle is 1.4 times the volume of the FNAB needles. Nonetheless, USeFNAB showed an increase in mass of 1.6 and 4.4 times compared to FNAB and CNB, respectively. Peculiar to salivary pleomorphic adenomas is a mix of tissue textures, a semiliquid mucinous component with few epithelial cells and a firmer epithelial cell component [23]. Furthermore, blood in the sample may distort the yield assessment. Therefore, mass may not be an optimal measurement for comparing the samples in an *in vivo* setting, rationalizing an evaluation of the sample area of the histologic sample as well. During the histological preparation, the blood was washed away prior to histological examination. The tissue area increased by 1.7 times with USeFNAB as compared to FNAB and by 3.4 times compared to CNB. The larger sample (*i.e*., larger area) provides a better opportunity to evaluate the histopathological features of the specimens. In addition, it enables the collection of more material, which can be used for ancillary studies such as immunohistochemistry, fluorescence *in situ* hybridization, and genetic analysis. These factors can lead to a more comprehensive analysis and, thus, a more accurate diagnosis while potentially reducing the need for repeated procedures. Additionally, the finding that a fine-needle biopsy can produce a higher sample yield compared to traditional sampling methods may be particularly advantageous in cases where CNB is contraindicated or limited by anatomical constraints. Moreover, because USeFNAB utilizes the needle of the same diameter as FNAB, it may be less invasive and cause fewer complications than CNB.

In this study, USeFNAB and FNAB provided suitable samples for both cytological and histological analysis from a single specimen, whereas CNB, as usual, provided only tissue construct for histological analysis. If USeFNAB proves capable of producing material for both types of analysis, it could offer pathologists the ability to assess both cellular characteristics and tissue architecture without additional steps required during the sample preparation process. Moreover, cytological and histological techniques are complementary in assessing tumor biology and facilitating the use of ancillary diagnostic techniques such as immunohistochemistry, electron microscopy, and molecular diagnostics [24]. Nonetheless, further investigation across different tissue types and various pathologies is needed.

Sample quality is critical alongside sample quantity with cellular content. In our series, the quality of cells obtained by both USeFNAB and FNAB remained adequate for diagnosis, with no damage in the cytological slides or histofragments detected as judged by blinded pathologists. In this study, single-hit CNB did not provide significantly better quality compared to USeFNAB or FNAB. In clinical practice, 1‒3 specimen cores are usually taken from a lesion with CNB. In our study setting, only one biopsy was taken with each needle sampling method, which can at least partly explain the smaller tissue area obtained with CNB. Although the quality of cells obtained by CNB was high, one specimen core represents only one location of the lesion. Multiple passes can overcome this issue, but require multiple needle penetrations, increasing the complexity, patient anxiety, and potential complications of the procedure. Especially in the case of a heterogeneous tumor, it is important that the sample represents the tumor from a large volume. Both USeFNAB and FNAB have, therefore, the advantage of utilizing the fanning technique to achieve this.

Due to the pilot study setting in the operation theater, the time points of each needle sampling technique were not measured. However, there were no noticeable differences in the sampling duration comparing USeFNAB, FNAB, and CNB. The operating radiologist found that sampling with USeFNAB did not notably differ technically from FNAB. Like FNAB, USeFNAB has the advantage of better tissue permeability compared to CNB using a thicker needle.

A number of limitations are present in this study, such as all three needle samples were taken consecutively within a 10-min span. It was not possible to distinguish whether any of the sample methods alone caused potential harm to the target lesion or the patient. In addition, immediately after the sampling, the patient underwent parotidectomy, which usually causes, among other things, both swelling and pain in the operated area [25]. Therefore, although no significant adverse events were reported, postoperative symptoms could not definitively be attributed to either the biopsies or subsequent surgery. The absence of patient feedback due to general anesthesia limits insights into USeFNAB-induced sensations, though previous studies suggest its invasive nature is comparable to FNAB using the same needle size. Another limitation was the restricted number of samples, and visually, the histological samples could be identified as obtained either with FNAB or USeFNAB, limiting objective blinded evaluation. While no significant adverse events were observed, the small cohort size and short follow-up period of this pilot study limit the generalizability of findings. The applicability of the study’s findings is also limited, as a pilot study with a limited sample size, combined with the exploratory aim of the study, adjusted analyses were not appropriate for the current analysis.

We minimized potential confounding factors by selecting only benign solid parotid gland tumors for the study, conducting the study in a general anesthesia setting, and using only one experienced radiologist to take the samples. This may have an undefined effect on the quality of all samples and the absence of typical nondiagnostic needle findings.

One failed calibration of USeFNAB in one patient caused the ultrasound to not activate during biopsy, thereby excluding the patient from the study.

To conclude, this first clinical study showed that USeFNAB offered improved yield in salivary gland tumors by 1.6 and 4.4 times compared to FNAB and CNB, respectively, while maintaining intact tissue quality and providing diagnostic samples in all the cases. Furthermore, USeFNAB was proven to be a feasible and safe technique within a clinical setting. The study did not reveal any factors that would prevent the implementation of USeFNAB in an outpatient clinic. Notably, USeFNAB’s potential extends beyond salivary gland tumors, warranting further research into its application for other tumor types and anatomical locations. As more data will be generated on the feasibility of USeFNAB, it may potentially be a good alternative to conventional needle sampling methods in future diagnostic practice.

## Supplementary information

**Additional file 1**: **Table S1** Pairwise comparisons between methods for histological quality. **Table S2** Histological quality scores per biopsy method assessed by the pathologists. **Table S3** Pairwise comparisons between methods for the sample mass collection. **Table S4** Pairwise comparisons between methods for the tissue area.

## Data Availability

The datasets used and/or analyzed during the current study are available from the corresponding authors upon reasonable request.

## Abbreviations

CNB: Core-needle biopsy
FNAB: Fine-needle aspiration biopsy
USeFNAB: Ultrasound-enhanced fine-needle aspiration biopsy
