# Supplementary material for: Ultrasound-enhanced fine-needle aspiration biopsy improves yield of solid benign parotid gland tumor tissue: a pilot study
Source: Eur Radiol Exp. 2026 Apr 2;10:41. doi: 10.1186/s41747-026-00707-0 (PMC13046893; doi:10.1186/s41747-026-00707-0)
Supplement: Supplementary file 1 — Additional file 1: Table S1 Pairwise comparisons between methods for histological quality. Table S2 Histological quality scores per biopsy method assessed by the pathologists. Table S3 Pairwise comparisons between methods for the sample mass collection. Table S4 Pairwise comparisons between methods for the tissue area. [file 41747_2026_707_MOESM1_ESM.pdf]

Ultrasound-enhanced fine-needle aspiration biopsy improves yield of solid benign parotid gland tumor tissue: a pilot study

ELECTRONIC SUPPLEMENTARY MATERIAL

Table S1 Pairwise comparisons between methods for histological quality

Method	Median (score)	Mean ± SD (score)	Mean difference *versus* USeFNAB ± SD [95% CI] (score)	Wilcoxon *p*-value	Rank-biserial correlation r_{rb}
USeFNAB	3.5	3.5 ± 0.5	–	–	–
FNAB	4	3.6 ± 0.5	-0.10 ± 0.32 [-0.33, 0.13]	1.000	-1.00
CNB	4	3.8 ± 0.4	-0.30 ± 0.48 [-0.65, -0.05]	0.250	-1.00

The results indicate a comparable tissue quality among the methods, with CNB and FNAB achieving slightly higher scores. However, no statistical significances were observed ($0.25 \leq p \leq 1.00$). *CI* Confidence interval, *CNB* Core needle biopsy, *FNAB* Fine-needle aspiration biopsy, *SD* Standard deviation, *USeFNAB* Ultrasound-enhanced fine-needle aspiration biopsy.

Table S2 Histological quality scores per biopsy method assessed by the pathologists

Method	% in category 0	% in category 1	% in category 2	% in category 3	% in category 4	Diagnostic samples (*n*, %)
USeFNAB	0	0	0	50	50	10 (100%)
FNAB	0	0	0	40	60	10 (100%)
CNB	0	0	0	20	80	10 (100%)

All methods scored as 3 or 4, with all reaching diagnostic. Quality levels 1, 2, 3 and 4 refer to 0–25%, 26–50%, 51–75% or 76–100% of the sample being intact. *CNB* Core needle biopsy, *FNAB* Fine-needle aspiration biopsy, *USeFNAB* Ultrasound-enhanced fine-needle aspiration biopsy.

Table S3 Pairwise comparisons between methods for the sample mass collection.

Method	Median (mg)	Mean ± SD (mg)	Mean difference *versus* USeFNAB ± SD [95% CI] (mg)	Wilcoxon *p*-value	Rank-biserial correlation r_{rb}
USeFNAB	24.05	25.24 ± 14.08	–	–	–
FNAB	12.55	15.98 ± 11.74	9.26 ± 12.41 [0.39, 18.13]	0.049	0.71
CNB	6.30	5.77 ± 1.90	19.47 ± 13.97 [9.47, 29.47]	0.002	1.00

USeFNAB collected, on average, 1.6 and 4.4 times more sample mass as compared to FNAB and CNB, respectively, with these differences being statistically significant. *CI* Confidence interval, *CNB* Core needle biopsy, *FNAB* Fine-needle aspiration biopsy, *SD* Standard deviation, *USeFNAB* Ultrasound-enhanced fine-needle aspiration biopsy.

Table S4 Pairwise comparisons between methods for the tissue area

Method	Median (mm^2)	Mean ± SD (mm^2)	Mean difference *versus* USeFNAB ± SD [95% CI] (mm^2)	Wilcoxon *p*-value	Rank-biserial correlation r_{rb}
USeFNAB	13.54	13.69 ± 8.47	–	–	–
FNAB	7.88	8.19 ± 5.73	5.50 ± 7.13 [0.40, 10.60]	0.049	0.71
CNB	3.82	4.06 ± 1.95	9.63 ± 7.94 [3.95, 15.31]	0.004	0.96

The results demonstrate an increase of the tissue area on the histological slide by 1.7 and 3.4 times when using USeFNAB compared to FNAB and CNB, respectively. These differences are statistically significant. *CI* Confidence interval, *CNB* Core needle biopsy, *FNAB* Fine-needle aspiration biopsy, *SD* Standard deviation, *USeFNAB* Ultrasound-enhanced fine-needle aspiration biopsy.
